# Early career interview: Hannah Wardill

**DOI:** 10.4155/fsoa-2019-0011

**Published:** 2019-04-12

**Authors:** Hannah R Wardill

**Affiliations:** 1 Research Fellow, the University of Adelaide, Australia & the University of Groningen, The Netherlands

**Keywords:** early career researcher, microbiome, mucositis, paediatrics, risk prediction, supportive oncology

Hannah Wardill was one of five finalists for the Future Science Early Career Research Award 2018. Read her interview to find out about her career, hopes for the future and advice to other early career researchers.

## Please tell us about your career history to-date

I completed my undergraduate degree in The Faculty of Health and Medical Sciences topping each of my three majors, before being awarded the Dean's Certificate of Undergraduate Merit. I then started honours in the School of Medicine in 2012, during which time I published two literature reviews and a primary research article. I was then named dux of my honours year and was given the prestigious honour of being Mace Bearer at my graduation. I then continued in the same research group, commencing my PhD in 2013.

During my PhD, I generated my own research and travel funding and was awarded the Florey Medical Research Foundation Doctor Chun Chung and Madam So Sau Lam Memorial Scholarship for PhD Excellence in Cancer Research. I presented my research at several national and international conferences including the Multinational Association for Supportive Care in Cancer (MASCC), the Australian Society for Medical Research and The Neuroimmunophysiology of the Gastrointestinal Tract Symposium. My work was recognized for its quality, novelty and impact, awarded numerous awards and I was named the International Young Investigator of the Year in 2016 at MASCC.

After completing my PhD, I was awarded Dean's Commendation for Doctoral Thesis Excellence and the University Medal for Doctoral Research Excellence; awards reserved for only a small handful of people. I was again given the privilege of being Mace Bearer at my doctorate graduation. In recognition of these achievements, I was also named one of three finalists in the SA Science Excellence Awards, and was named SA Young Achiever of the Year (Science/Technology).

In 2016, I commenced my first postdoctoral research position at the South Australian Health and Medical Research Institute, after being personally selected by the laboratory's PI. During this time, I came to realize my thirst for independence and goal of establishing my own research group. I subsequently applied for a National Health and Medical Research Committee (NHMRC) fellowship, in which I was successful; the pinnacle of my research career to-date. In 2018, I moved to The Netherlands to start my NHMRC CJ Martin Biomedical Research Fellowship in pediatric oncology. During this time, I have established myself as an emerging and driven ECR in the field of supportive oncology. In recognition of this, I have been appointed to several leadership positions within the Multinational Association for Supportive Care in Cancer (MASCC), an international organisation dedicated to all aspects of supportive oncology. I am currently leading the update of the clinical practice guidelines for the management of mucositis secondary to cancer treatment, a task that involves coordinating the systematic review of literature and dissemination of clinical practice guidelines. I am also the Chair of the Social Media Committee, a new initiative adopted by MASCC in 2018 to engage their members with people affected by cancer.

## What made you choose a career in your field?

I have always shown curiosity for the world around me, and been driven to solve problems and intrigued by ingenious solutions. As such, a career in science was always suited for me. Even as a young child, I was involved in many science programs, awarded the Oliphant Science Award in 2005. When deciding which field I wanted to pursue, medical sciences was a clear choice as I have always had an insatiable quest to solve problems relating to human disease. Originally, I was interested in pure cancer biology reflecting the prevalence of cancer in my own family, and the presence of a heritable mutation in my extended family predisposing to bowel cancer (Lynch Syndrome/HNPCC). However, in an undergraduate project I became exposed to the work being conducted in supportive cancer care at the University of Adelaide. In addition to being completely inspired by my supervisors at the time, the research field resonated with me because I felt I could have a genuine impact on peoples’ lives undergoing cancer treatment. I was driven to challenge the view that side effects of chemotherapy are ‘unavoidable’ or a ‘necessary evil’ that patients must endure in order to achieve remission. I am highly competitive, and I saw this as a challenge to eliminate some of the most debilitating side effects, thus making the lives of people undergoing treatment not only tolerable, but also enjoyable. Through my involvement with the patient advocacy group, Cancer Voices, I have had the opportunity to meet with people that have either experienced these side effects, or in fact benefited directly from research in my laboratory. This has been an excellent motivator to continue this line of research as it has genuine and meaningful impacts on not only the patient, but their family and the wider community.

## Describe the main highlights of your career so far

The key highlights of my career to date are multifactorial. It was a great honor to be selected as Mace Bearer at both my undergraduate and doctorate graduation ceremonies. This is a long-standing tradition at the University of Adelaide, and it was incredibly humbling to be given that honor twice.

It was also an extremely humbling experience to be recognized for my work by several organisations/foundations including the SA Young Achiever of the Year and the SA Science Excellence Awards. To be recognized alongside the caliber of my fellow recipients/awardees highlighted to me the importance of my work and the impact of its outcomes. Being named the SA Young Achiever and finalist in the SA Science Excellence Awards put my research on a community-based platform, and the benefits I gained from this were immense. In the same year I was named the International MASCC Young Investigator of the Year, received Dean's Commendation for Doctoral Thesis Excellence and the University Medal for Doctoral Research Excellence. These awards were extremely encouraging to receive as they reinforced the quality, novelty and impact of my research output during my PhD. Most recently, I was named a finalist in the You Can Innovate Awards, a grant assessed by young cancer patients. It was encouraging to see that my research resonated with this cohort.

On a personal level, I have also found working with the patient advocacy group Cancer Voices an extreme highlight. The feedback and encouragement received from current patients, cancer survivors and people affected by cancer has been extremely rewarding and serves as a constant reminder of why I have chosen such a challenging career path.

Scientifically, my career highlights have certainly been based around traditional metrics of scientific success. I am extremely proud of my publication track record, with 32 peer-reviewed publications to date. The pinnacle for me has certainly been securing my NHMRC CJ Martin Biomedical Research Fellowship. This award is offered only to a limited number of people of outstanding ability who wish to make biomedical research a significant component of their career. In the same few months, I was also awarded significant funding from the Royal Adelaide Hospital and Hospital Research Foundation to conduct my FMT clinical trial. This was extremely important in enabling me to establish my own research niche and independence, and reinforced the validity and impact of my research aspirations. With this came an increased reputation as an ‘expert’ in my field, resulting in heightened media engagement and science communication. Although not traditionally viewed as a marker of scientific success, being asked to provide expert opinion on radio and news channels has been a rewarding experience for me as I am passionate about the dissemination of scientific literature and encouraging scientific discovery in the lay community. Particular highlights for me were appearing on a large, mainstream news channel and writing for The Conversation.

Finally, it has also been a significant career highlight to be recognized for my dedication to supportive oncology by the Multinational Association for Supportive Care in Cancer (MASCC), undoubtedly the key leader in my field of research. In 2017 I was appointed as the Section Co-Chair for the update of the clinical practice guidelines for the management of mucositis, and Chair of the Social Media Committee. These roles come with significant responsibility, reinforcing my contribution to the field and leadership skills.

## Describe the most difficult challenge you have faced & how you overcame it

One major challenge I have faced during my research career has been issues relating to gender equity and diversity. To date, I have not been directly impacted by this; however, I am acutely aware of the challenges that face women in STEMM subjects. This is particularly the case for women in science, at the early-mid career level, with substantial decreases in the amount of women progressing to higher positions. This ultimately impacts on their ability to secure grant funding, and hence impairs their career progression. These inherent disparities have driven me to become actively involved in a number of committees and programs (e.g., #BeBoldforChange, Women in Science and Engineering, #STEMSelfie) that aim to empower women in STEMM and engage younger girls with science.

## How do you feel you have impacted your field?

My past and current research endeavors have significantly contributed to the field of support care in cancer via several distinct pathways:

### Advancement of fundamental knowledge

A large portion of my PhD research focused on the innate immune interactions underpinning chemotherapy-induced gastrointestinal toxicity. My work contributed significantly to our understanding of the pathobiological mechanisms responsible for its development. Importantly, this contribution was recognized, and my findings incorporated into the current pathobiological model used to describe gastrointestinal toxicity. These findings also led to the development of new interventions to improve the outcomes of cancer care.

### New method of risk prediction

In addition to understanding the pathobiology, my research has contributed significantly to the concept of risk prediction. To date, the majority of risk prediction research has centered on genomic profiling, with particular SNPs related to treatment efficacy and toxicity. This is a justified approach, however many of the identified SNPs are only expressed in a very small portion of the general population and are unable to be modified. My research was the first to suggest that an individual's microbiome may be useful in determining their treatment outcomes. The microbiome is an attractive method of risk prediction, as it is highly heterogeneous among people, and highly plastic, enabling methods of risk mitigation to be implemented. Furthermore, by comprehensively characterizing the microbiome of people that respond optimally to cancer therapy (i.e. high efficacy, low toxicity), we can design interventions that target specific mechanisms of the pathobiology. This is particularly important for the microbiome, as there is a tendency to use generalized probiotics without robust justification. By understanding how an individual's pretreatment microbiome regulates their response to treatment, there is the distinct possibility of providing personalized and tailored microbial interventions that will enhance treatment outcomes.

### Social media strategies

In addition to my contribution to the mechanistic understanding, management and modelling of gastrointestinal toxicity, I am driven to always improve current practices. One of the major challenges in supportive oncology is the collection of patient-reported outcomes, with diarrhea particularly difficult to discuss with patients and thus almost impossible to quantify. In collaboration with MASCC, I am leading the development of a mobile app for the collection of patient-reported outcomes in a structured manner. Although in its early stages of design, this app will promote regular data entry in a user-friendly interface to ensure robust data acquisition based on images, and clear descriptions. This initiative has been supported by MASCC, highlighting its value to the field of supportive oncology.

## What are your aims for the future?

My key area of research interest is now the potential to predict an individual's response to treatment, thus enabling personalized approaches to their management. I am currently developing a risk stratification model based on the unique features of an individual's gut bacteria, their genetic variants in immune signalling pathways and their lifestyle/environmental factors, with the aim of providing a comprehensive analysis of their risk of developing severe gut side effects during treatment.

I also plan to extend this model to not only predict gastrointestinal toxicities (diarrhea), but also extra-intestinal side effects of chemotherapy such as cognitive dysfunction. Cognitive dysfunction is a chronic side effect of chemotherapy that persists long after treatment ends. Patients present with a myriad of complaints including concentration difficulties, low processing speeds, learning difficulties and memory deficits which prevent them from being able to return to their previous level of social and academic interaction. In children, cognitive dysfunction is critically important, and poorly managed. Given the chronicity of these symptoms, my research has the potential to enhance the lives of children with cancer, preventing long-term deficits in learning, memory and concentration. This will ensure that cancer treatment does not place children at a disadvantage following remission.

I also hope to expand this model to incorporate tumor response, aiming to predict how well an individual's tumor will respond to chemotherapy, thus predicting chances of remission. This will have relevance to gastrointestinal cancers, as the gut microbiome is a well-described component of tumor development and growth. Hence, analyzing the gut microbiome before treatment may shed light on how a patient is likely to respond to treatment.

Given my keen interest in science communication, I also hope to continue engaging with the general public via mainstream media outlets and my own personal blog. Upon my return to Adelaide in 2020, I hope to be selected to take part in the Superstars of STEM initiative to improve my public outreach and simultaneously become a role model for young women interested in science.

On a broader scale, my ultimate goal is to head my own research laboratory underpinned by a program of translatable research projects related to supportive oncology. I hope to supervise numerous students, providing a rich environment for scientific and personal growth, access to world class facilities and engagement with key clinicians to promote a bench-to-bedside approach.

## What advice do you have for those hoping to win the award in the future?

I believe the key to success is ensuring you continue to build a multifactorial skill set, in which you draw inspiration from those around you. At the core should be a scientific career built upon traditional metrics such as publication track record and funding success; however surrounding that should be a diverse mix of community engagement, scientific outreach and leadership experience. These activities not only allow you to disseminate your research to a wider audience, but they also help you recognized the impact of your work and the key areas of need in your specific research field.

